# Metabolic syndrome and different obesity phenotypes in the elderly women population: Iran’s Health System on aging

**DOI:** 10.22088/cjim.9.3.252

**Published:** 2018

**Authors:** Afsaneh Bakhtiari, Karimollah Hajian-Tilaki, Azita Ghanbarpour

**Affiliations:** 1 Infertility and Reproductive Health Research Center, Health Research Institute, Babol University of Medical Sciences, Babol, Iran; 2 Social Determinants of Health Research Center, Health Research Institute, Babol University of Medical Sciences, Babol, Iran; 3 Clinical Research Development unit of Ayatollah Rouhani Hospital, Babol University of Medical Sciences, Babol, Iran

**Keywords:** Obesity phenotypes, Elderly women, Metabolic syndrome, Prevalence

## Abstract

**Background:**

Current literature has been focused on types of obesity with normal BMI (body mass index), but metabolically unhealthy.This study evaluates the prevalence of metabolical phenotypes of obesity. We also identified the best obesity index in predicting the components of metabolic syndrome (MetS).

**Methods:**

A cross-sectional** s**tudy has been conducted on 164 women over 60 years. Anthropometric parameters, body fat percentage (%BF), and biologic criteria were measured to assess the types of obesity. Unhealthy metabolic was defined by modified Adult Treatment Panel III, and obesity based on BMI≥25.ANOVA and logistic regression were utilized for the association of MetS components and obesity phenotypes, and linear regression logistic for finding the best MetS related obesity index.

**Results:**

The prevalence of metabolically unhealthy was 45.7%, out of which 33.3% was among the individuals with normal BMI.Logistic regression has shown that triglyceride (TG) (OR=3.30, p<0.001) and high density lipoprotein (HDL-C) (OR=2.15,p<0.01) was independently related to metabolically healthy and normal weight(MHNW) phenotype. Moreover, TG (OR=3.92,p<0.001), HDL-C (OR=2.18,p<0.001), fasting blood glucose(FBG) (OR=1.73,p<0.01) and waist circumference(WC) (OR=3.18,p<0.001) are correlated significantly with metabolically unhealthy and overweight/obese (MUO) and also TG (OR=2.88,p<0.001) and WC (OR=2.67,p<0.001) with metabolically unhealthy and overweight/obese(MHO).WC followed by %body fat (BF) showed to be highly correlated with the prognosis of MetS components.

**Conclusions:**

There is a high prevalence of unhealthy metabolic among the elderly women,even with normal weight.There were different associations between MetS components and various obesity phenotypes.TG was the most powerful indicator for the prognosis of unhealthy metabolic phenotypes which was independently correlated with the WC, %BF and BMI.

Obesity consists of different subtypes with different metabolic profiles. Based on body mass index (BMI) and metabolic status, obesity is categorized into four groups which includes metabolically healthy and normal weight (MHNW), metabolically unhealthy and normal weight (MUNW), metabolically healthy and overweight/obese (MHO), metabolically unhealthy and overweight/obese (MUO) (1). MHNW is a subset of individuals with normal weight based on BMI and a high body fat content of visceral fat. These individuals have shown an increase in the risk of cardiovascular disease (CVD) (2). On the other hand, within the obese population, the small portion (10-25%) who do not display metabolic disturbances are termed MHO (3). 

Different mechanisms behind the different obesity phenotypes include genetic, socioeconomic and behavioral factors, some of which may be modifiable (4). Studies also have shown that obesity-related metabolic phenotype changes over time. They found that the transition from menopause to aging is accompanied by changes in metabolic status in individuals with different obesity phenotypes (5-7). Elderly people in particular are faced with considerable changes in body composition and also redistribution fat in the body. Therefore, aging can put people at an increased risk of developing central obesity and metabolic disorders as a result of multiple age-related physiological mechanisms (8). This is because excess total body fat, specifically intra-abdominal fat that is associated with CVD risk factors, such as insulin resistance, diabetes, hypertension, and dyslipidemia, are all components of metabolic syndrome (MetS) (9,10).

Statistics are indicative of an increasing trend in the prevalence of obesity and MetS in Iran (5, 6, 11, 12) and other countries (1, 7, 9, 10, 13). Unhealthy metabolic prevalence among individuals with normal weight and overweight varies in different studies. In one study, the prevalence of unhealthy metabolic was reported 67% for elderly individuals with normal weight compared to 81% among overweight of the same age group (13). Studies on different obesity subtypes are very limited in elderly women in Iran. A study conducted on individuals of the age range of 20-72 in Qazvin, Iran demonstrated the unhealthy metabolic phenotype prevalence as 33.8% for normal weight men, and 39.8% for women (11). 

This is while its prevalence and correlation with various obesity phenotypes are still unclear. Moreover, some of the studies demonstrated a relation between blood pressure, HDL-C, and triglyceride only with a single phenotype of obesity (for example MHUNW or MHO) (2, 11, 13). Others have examined the relationship between the phenotype of obesity with risk of development of diabetes, CVD or cancer in different age groups and demographics (1, 3, 12), but the association between MetS and all of the obesity phenotypes together, especially in elderly people is less investigated.

We believe that our understanding about the prevalence of various obesity phenotypes, and their correlation with MetS components can help us better in our decision making about the elderly individuals’ health.  Thus, this study determines the prevalence of different obesity phenotypes and measures association between them with MetS components, and finally, identifies the best index of obesity for predicting MetS components in elderly women in Babol, Iran (in 2011). 

## Methods

**Subject: **This cross-sectional study was conducted in the rural areas of the central part of Babol in Mazandaran, Iran. The participants were recruited through community advertisement by poster.  The posters were distributed in the rural health clinics affiliated with the Babol University of Medical Sciences, Iran. *306 volunteers were enrolled*. After the exclusion of 142 individuals in terms of exclusion criteria and lack of consent to participate in the study, a total number of 164 eligible women 60 years or over with lipid and/or glucose disorders, abdominal obesity or hypertension remained for the study. With the presumption of prevalence of MetS (40%), the allocated sample size could estimate the prevalence of MetS with confidence level of 95% and marginal error of 0.07.

All the participants were free of kidney, liver, thyroid, cardiovascular disease, cancer or any other chronic diseases and that were confirmed through history and medical reports and documents. They also did not receive any medications for diabetes, hyperlipidemia and hypertension. 

 A questionnaire on demographic characteristics was completed by the researcher for each participant based on a face-to-face interview. The study protocol was approved by the Ethics Committee of Babol University of Medical Sciences (NO.:MUBABOL.REC.1388.1). All participants provided an informed written and signed consent form.

**Anthropometric Measurements: **Participants’ height was measured using a stadiometer without shoes to the nearest 0.5 cm. Weight, body fat percentage (%BF), and BMI were measured with composition analyzer (Omron HBF-306 body fat analyzer). BMI and %BF values were defined according to the World Health Organization criteria (1). Waist circumference (WC) was measured at the mid-point between the iliac crest and the lowest rib and hip at the widest point. BMI was subdivided into normal weight if BMI was less than 25 and overweight/obese if BMI was 25 or greater. The combination of overweight (25–29.9 kg/m^2^), and obese (≥30 kg/m^2^) in one group overweight/obese was done to increase statistical power. In addition, abnormal %BF was defined as %BF over the gender-specific tertile (38% for women and 26% for men).

**Metabolic healthy: **A modified Adult Treatment Panel (ATP) III definition of MetS criteria was used to define metabolically healthy (<3 risk characteristics), including WC>80 cm; HDL-C <50 mg/dL; triglyceride ≥150 mg/dL; fasting blood glucose ≥100 mg/dL; and systolic blood pressure ≥130 mmHg and diastolic ≥85 mmHg. Participants received specific instruction prior to their blood sample including no food or beverage (except water) for 10-12 hours overnight fasting. 10 mL of venous blood was drawn (at 7-8 am) and collected into test tubes. Serum was separated by centrifugation within 15 minutes of collection. The total cholesterol (TC) and TG levels were measured using an Elitech kit from France; LDL-C, HDL-C, and FBG were assayed on a Mindray-BS300 chemistry autoanalyzer (Mindray-BS300, Nanshan, Shenzhen, China). Systolic and diastolic blood pressures were assessed using the ALPK2 aneroid model sphygmomanometer, (Tanaka Sangyo, Co. LTD. Tokyo, Japan) by the researcher, twice on the right arm after patients were instructed to remain seated for 10 minutes;. The average of two seated systolic and diastolic blood pressure measurements were used for data analysis. 

**Statistical analysis: **Normality of variable of interest was tested by Kolmogorov–Smirnov. The analysis compared the four metabolic and obesity phenotypes using ANOVA for normally distributed continuous variables. Tukey test served as a post-hoc test to perform a pairwise comparison of the means to see where the significant difference lies. The independent association of the MetS components with different obesity phenotypes was assessed by logistic regression. The MetS components were classified as normal or abnormal as defined by ATP III. The participants with normal status were considered as reference. Also, multiple linear regressions were used to evaluate the prediction of metabolic biomarkers of CVD by %BF and anthropometric measurements. The two-tailed p-value less than 0.05 was considered significant. The statistical analyses were all performed using SPSS for windows (Version 20). 

## Results

The study was performed on elderly women with a BMI 26.8±0.44, mean age of 64.2±3.8 years and mean age of menopause 45.9±4.3 years. Among the 164 subjects, 75 (45.7%) individuals were diagnosed with unhealthy metabolic which includes 25 (15.2%) MHNW and 50 (30.5%) MUO (Table 1).

**Table 1:** Physical and biochemical characteristics in the elderly women (mean ± SD)

	**Overall** ** n=164**	**MHNW** **(n=43)**	**MUNW** **(n=25)**	**MHO** **(n=46)**	**MUO** **(n=50)**
BMI (kg/m^2^)	26.8±0.44	24.2±0.54	24.8±0.59	28.4±0.68^a^	29.8±0.81^a^
WC (cm)	90.75±6.8	81.2±3.2	83.6±5.4	96.8±8.5^a^	101.4±9.5^a^
Body fat (%)	40.3±1.48	32.3±0.62	39.2±0.67^b^	40.4±0.96^b^	44.8±1.96^b^
SBP (mmHg)	137±0.78	134±0.36	135±0.44	131±0.47	139±0.56
DBP (mmHg)	73±1.3	73±0.90	71±1.34	74±1.4	76±1.38
TC (mg/dL)	205.6±34	213.2±30	231.6±23	217.8±26	226.2±25
LDL-C (mg/dL)	121±30	125±28	138±20	127±23	142±24
HDL-C (mg/dL)	51±4.5	64±5.48	49±3.6 c	59±5.2	44±4.01 c
TG (mg/dL)	177±21	150±32	196±21 c	164±29^d^	212±26 c
FBG (mg/dL)	97.8±2.2	93±2.18	102.8±2.01 c	95±2.34	104.5±2.49 c
TG/HDL-C ratio	3.38±0.27	2.56±0.34	4±0.25^c^	2.42±0.21	4.90±0.28^c^

MHNW: metabolically healthy and normal weight; MUNW: metabolically unhealthy and normal weight; MHO: metabolically healthy and overweight/obese; MUO: metabolically unhealthy and overweight/obese; BMI: body mass index; WC: waist circumference; SBP: systolic blood pressure; DBP: diastolic blood pressure; TC: total cholesterol; LDL-C: low density lipoprotein; HDL-C: high density lipoprotein; TG: triglyceride; FBG: fasting blood glucose

a . p˂0.001 versus other groups

b . p˂0.001 versus MHNW group

c . p˂0.001 versus other groups

d . p˂0.01 versus MHNW group

The most common components of MetS were higher WC (in more than half of the individuals), followed by low HDL-C and high TG levels (in more than 40% of the individuals). 

There was no meaningful difference among the 4 metabolic cluster groups in terms of demographics and medical history. Post-hoc test depicted substantially higher TG, FBG, TG/HDL-C levels, and lower levels of HDL-C among unhealthy metabolic groups compared to the other groups. WC displayed no significant difference between the two groups of MUNW and MHNW (Table 1), in contrast to %BF (p<0.0001). Evaluation of the independent effect of each MetS component on different obesity phenotypes compared to the MHNW group (as reference group) showed that TG (OR=3.30, p<0.001) and HDL-C (OR=2.15, p<0.01) were independently related to MUNW phenotype. Moreover, TG (OR=3.92, p<0.001), HDL-C (OR=2.18, p<0.001), FBG (OR=1.73, p<0.01) and WC (OR=3.18, p<0.001) are correlated with MUO and also TG (OR= 2.88, p<0.001) and WC (OR= 2.67, p<0.001) with MHO (Table 2).

The relationship between indexes of obesity and MetS components revealed that WC was an independent predictor for TG, FBG, HDL-C and TG/HDL-C, respectively. In the same way, %BF was an independent predictor for TG, FBG, as well as BMI for TG (Table 3).

**Table 2:** Multiple Logistic regression analysis of the MetS components and different phenotypes of obesity

**Predictor variables**	**MUNW**		**MHO**		**MUO**	
	**Unadjusted** **OR (95% CI)**	**Adjusted** **OR (95% CI)**	**Unadjusted** **OR (95% CI)**	**Adjusted** **OR (95% CI)**	**Unadjusted** **OR (95% CI)**	**Adjusted** **OR (95% CI)**
WC^a^	1.94 (0.71-3.15)	1.86 (0.55-4.24)	3.15 (1.98-3.65)^1^	2.67 (2.13-4.67)^1^	3.25 (2.01-5.42)^1^	3.18 (2.11-4.32)^1^
TG^b^	3.37 (1.46-5.28)^1^	3.30 (2.67-5.45)^1^	2.91 (1.49-4.33)^2^	2.88 (2.12-3.65)^1^	3.88 (2.68-6.87)^1^	3.92 (2.28-5.17)^1^
SBP^c^	1.80 (0.94-2.32)	2.34 (0.98-4.34)	1.32 (0.66-3.18)	1.58 (0.60-2.23)	1.14 (0.39-3.62)	0.87 (0.25-3.44)
DBP^d^	0.46 (0.35-1.40)	1.22 (0.45-2.56)	0.26 (0.63-2.82)	1.06 (0.43-2.29)	0.82 (0.47-3.42)	0.76 (0.57-2.88)
HDL-C^e^	1.36 (1.00-2.12)^2^	2.15 (1.29-3.19)^2^	1.02 (0.45-2.36)	0.92 (0.85-2.71)	1.68 (1.08-2.98)^2^	2.18 (1.98-3.72)^1^
FBG^f^	1.92 (1.36-4.44)^2^	1.14 (0.51-3.89)	0.80 (0.22-4.40)	1.49 (0.72-3.98)	1.14 (1.08-4.85)^2^	1.73 (1.26-4.65)^2^

Note.WC, waist circumference; TG, triglyceride; SBP, systolic blood pressure; DBP, diastolic blood pressure; HDL-C, high density lipoprotein; FBG, fasting blood glucose; MUNW, metabolically unhealthy and normal weight; MHO, metabolically healthy and overweight/obese; MUO, metabolically unhealthy and overweight/obese.

Components of the MetS were defined as abnormal vs. normal. Moreover, different phenotypes of obesity were compared with MHNW.

a waist circumference˃80cm

b TGs ≥150 mg/dL

c HDL-C <50 mg/dL

d Systolic blood pressure ≥130 mmHg

e Diastolic blood pressure ≥85 mmHg

f fasting blood glucose ≥100 mg/dL

1 . P value˂0.001

2 . P value <0.01

**Table 3:** Multiple linear regression coefficient of %BF, WC and BMI (independent variables) with metabolic syndrome components (dependent variables)

**Dependent Variables**	**%BF**		**WC**		**BMI**	
	**Unstandardized coefficients** **B (SE)**	**Standardized coefficients** **β**	**Unstandardized coefficients** **B (SE)**	**Standardized coefficients** **β**	**Unstandardized coefficients** **B (SE)**	**Standardized coefficients** **β**
TC(mg/dL)	0.391 (0.12)	0.079	0.283 (0.016)	0.13	0.150 (0.450)	0.080
LDL-C(mg/dL)	0.016 (0.026)	0.064	0.022 (0.072)	0.06	0.06 (0.552)	0.017
HDL-C (mg/dL)	-0.293 (0.37)	-0.156	-0.430 (0.004)	-0.29^2^	-0.034 (0.251)	-0.135
TG (mg/dL)	0.634 (0.06)	0.334^1^	0.570 (0.092)	0.55^1^	0.159 (0.084)	0.24^2^
FBG (mg/dL)	0.231 (0.07)	0.201^2^	0.37 (0.0.87)	0.34^1^	0.056 (0.622)	0.120
TG/HDL-C	0.084 (0.29)	0.012	0.064 (0.004)	0.23^2^	0.014 (0.363)	0.073
SBP (mm/Hg)	0.003 (0.038)	0.026	0.002 (0.003)	0.026	0.064 (0.165)	0.105
DBP(mm/Hg)	0.005 (0.014)	0.029	0.001 (0.005)	0.052	0.04 (0.065)	0.021

Note.TC, total cholesterol; LDL-C, low density lipoprotein; HDL-C, high density lipoprotein; TG, triglyceride; SBP, systolic blood pressure; DBP, diastolic blood pressure; BF, body fat percentage; WC, waist circumference; BMI, body mass index.

1 . P value˂0.001

2 . P value <0.01

## Discussion

The study revealed that the prevalence of unhealthy metabolic was 45.7% in elderly women, with 36.8% among individuals with normal weight compared to 52.1% among overweight individuals. Roberson et al. (2014) also reported the prevalence of unhealthy metabolic as 67% for elderly individuals (80 years and older) with normal weight compared to 81% among overweight of the same age group, with marginal difference of 14% (13), which was similar to the findings of the present study (i.e., 36.8% versus 52.1%, with 15.3% difference). Unhealthy metabolic prevalence among elderly individuals with normal weight and overweight varies in different studies. For example, in one study, it has a prevalence rate of 22% among overweight, and 56% in normal weight individuals (14). 

There are no studies about MUNW of elderly women in Iran. A study in Iran, Ghazvin, conducted on individuals with age range of 20-72 demonstrated the unhealthy metabolic phenotype prevalence as 33.8% for normal weight men, and 39.8% for women. In this study, the lower limit of top quintile of HOMA-IR values was considered the unhealthy metabolic diagnostic criteria (11). 

These differences might partially be due to ethnicity variations causing metabolic disorders and different obesity phenotypes. Furthermore, definition of obesity based on BMI or excess body fat might be another factor also causing the differences (15). On the other hand, there is no general agreement in terms of excess adiposity. Researches introduced various cut-off points for defining the %BF among the elderly population (35-38% in women, and 24-26% in men) (1, 16, 17). Moreover, various studies utilized varying criteria for diagnosing the unhealthy metabolic; some of HOMA-IR amounts within the highest quartile ratio or more than 1.69 (18), some others use the ATP III criteria for Mets terminology (12). Recently, Lee et al. (2015) have utilized the TYG indicator (triglyceride x FBG) for MUNW identification (19). At anyrate, more studies are required to determine the obesity phenotypes in elderly population of Iran. 

We found that the amounts of TG, HDL-C, TG/HDL-C, and FBC have meaningful differences among unhealthy metabolic groups when compared to other groups. Previous studies also reported a higher prevalence of MetS and its components with the increase of %BF among normal weight individuals (2-3) as well as individuals with overweight/ obesity (14, 16). Tsou study (2012) on MetS prevalence among the elderly population of Taiwan with MUNW phenotype, also demonstrated a relatively high MetS prevalence in both groups of normal weight and overweight/obese (20). Conducted studies in Iran also depicted an increasing trend (4 times higher) of MetS prevalence among Tehran adults with normal weight (21, 22). As a CVD risk factor, MUNW is linked to adiposity concept. This is further discussed in a way that in MUNW, lean mass decreases and the fat mass rises while the BMI is within the normal range. This situation introduces the concept of sarcopenic obesity among the elderly population (3). Therefore, metabolic disorders with MUNW, like MetS, are associated with more complications in the elderly individuals (23). Jeans et al. (2014) demonstrated that sarcopenic obesity is clearly accompanied by the increased risk of MetS among the elderly individuals compared to individuals without sarcopenic obesity (3). In clinical settings, these individuals are not usually regarded as the cases with obesity or higher CVD risk, and therefore, are missed. 

Individuals with MHO are the counterpoints of these individuals. Recent studies have shown that these individuals are known as obese with high BMI, and huge subcutaneous fat storage in legs, hips, and buttock areas. These individuals have lower risk of CVD. Two longitudinal studies in Tehran, Iran showed that individuals with normal weight but dysmetabolic status are at greater risk for CVD than the healthy obese individuals (5, 22). However, some studies showed an increased CVD risk in MHO, when compared to the individuals with normal BMI and %BF (24, 25). The current research results demonstrated that WC and TG were independent risk factors for MHO groups when compared to the MHNW group. A recent longitudinal study (2015) of 2368 people in America has indicated that MHO might be an unstable status, and throughout time, might develop into multiple metabolic abnormalities (26). 

In the present study, when we evaluated the independent effects of Mets components on different obesity phenotypes we found that TG is the strongest independent predictors of unhealthy metabolic phenotypes after WC and HDL-C. High level of TG has been reported among individuals with high risk of CVD (11, 12) but its correlation with various obesity phenotypes especially among elderly population has not been studied very thoroughly. Hashemipour et al. (2015) demonstrated that among the different Mets components, TG was the only component which is solely related to insulin resistance in women with MUNW (11). Hypertriglyceridemia is the key feature of the MetS, and is linked with CVD. Elevated serum TG in older women may be considered as an indicator of enhanced TG-rich VLDL-C, which is particularly atherogenic. Additionally, elevated TG among women has been shown to generate greater atherogenic significance than that of men. In obese women with excess deep abdominal fat and also in women with increased %BF without overweight could be responsible for the reduction in plasma HDL-C levels (27). Lee et al. (2014) have recently introduced TG measurement along with FBG (TYG indicator) as useful indicator in MUNW definition (19). In the current study, WC, followed by %BF, depicted the highest correlation for prediction of the presence of MetS components among the elderly women. This is consistent with Charipour’s findings (2014) in the elderly men population of Isfahan, Iran (28). 

They found the WC as the best obesity indicator for the diagnosis of MetS in this group. Their previous study (2013), about finding the best indicator for MetS diagnosis regardless of age and sex also showed similar results (29). Many past studies also indicated WC and WHR as the most powerful instruments of Mets prognosis (30-32). Seo et al. (2009) also demonstrated WC at a competing level with visceral fat abdomen as powerful indicator for identifying elderly individuals at risk of MetS (33). Some researchers suggest that the WC, WHR and BMI are all equally useful indicators for the identification of metabolic abnormalities (34, 35). At any rate, the best obesity index for predicting CVD risk differs in various populations (36). 

Our study has some limitations. First, this is a cross-sectional study which does not provide proof for a temporal relationship. Besides, we took BMI>25 as the obesity index among elderly individuals; this cutoff value may not be suitable for elderly individuals. Moreover, further studies are required to determine the BMI- cutoff value for obesity definition in Iranian elderly women. Furthermore, this study may not cover the entire population of elderly women in the studied villages, thus disrupting the generalization of the results. However, it should be noted that given the limited number of women over the age of 60 in the studied areas and the good coverage of the population by health care providers, it can confidently be found that, except for women with health problems, the rest of the subjects entered the study.

The strong points of the current study included participants` relative importance of homogeneity with respect to age, sex, lifestyles and health condition and that no one was under the treatment of glycemic, lipid-lowering or antihypertensive drugs; this makes the results reliable. 

In conclusion there were different associations between MetS components and various obesity phenotypes. The most unfavorable metabolic profile was related to MUO followed by MUNW. MHO group although metabolically healthy, showed a significant difference in some MetS criteria when compared to MHNW. This supports the previous studies findings in terms of possible increased risk of metabolic disorders in the future among this group. Due to the existing inconsistencies, further studies are required in this respect, anyway. Likewise, TG was introduced as the most powerful indicator for prognosis of unhealthy metabolic phenotypes which was independently correlated with the WC, %BF and BMI. Hence, enough attention must be given to MetS and resulting complications in elderly women with elevated triglyceride levels and even among normal weight individuals. 
